# The amyloid precursor protein: beyond amyloid

**DOI:** 10.1186/1750-1326-1-5

**Published:** 2006-07-03

**Authors:** Hui Zheng, Edward H Koo

**Affiliations:** 1 Huffington Center on Aging and Department of Molecular & Human Genetics, Baylor College of Medicine, Houston, TX 77030, USA; 2 Department of Neurosciences, University of California, San Diego, La Jolla, CA 92093, USA

## Abstract

The amyloid precursor protein (APP) takes a central position in Alzheimer's disease (AD) pathogenesis: APP processing generates the β-amyloid (Aβ) peptides, which are deposited as the amyloid plaques in brains of AD individuals; Point mutations and duplications of APP are causal for a subset of early onset of familial Alzheimer's disease (FAD). Not surprisingly, the production and pathogenic effect of Aβ has been the central focus in AD field. Nevertheless, the biological properties of APP have also been the subject of intense investigation since its identification nearly 20 years ago as it demonstrates a number of interesting putative physiological roles. Several attractive models of APP function have been put forward recently based on in vitro biochemical studies. Genetic analyses of gain- and loss-of-function mutants in *Drosophila *and mouse have also revealed important insights into its biological activities in vivo. This article will review the current understanding of APP physiological functions.

## Background

Alzheimer's disease (AD) is the most common cause of dementia occurring in the elderly. It is characterized pathologically by the deposition of β-amyloid plaques, the accumulation of neurofibrillary tangles and loss of neurons and synapses in selected areas of the brain. The β-amyloid plaques are extracellular deposits of heterogeneous substances of which the major components are 40 to 42 amino acid peptides referred to as β-amyloid peptides (Aβ) that are derived by proteolytic cleavages of the amyloid precursor protein (APP). Liberation of Aβ from APP requires the action of β- and γ-secretases which process APP at amino-terminus and carboxyl-terminus of the Aβ sequence, respectively (reviewed in [1,2]). Approximately 5–10% of AD cases are familial and cosegregate with autosomal dominant inheritance of mutations in *APP *[3] and two homologous molecules, presenilin 1 (*PS1*) [4] and presenilin 2 (*PS2*) [5] (up-to-date genetic mutations can be found at ). PS1 or PS2 is a necessary component of the high molecular weight complex indispensable for γ-secretase processing of APP. The familial AD (FAD) mutations in the presenilin genes are known to subtly alter the γ-secretase activity to perturb APP proteolysis (reviewed in [6]). This biochemical and genetic evidence places APP and its processing steps in a central position in AD research.

This review focuses on the physiological properties of APP. We start with a general overview of APP, including its family members, expression patterns and processing characteristics. Because APP consists of multiple structural and functional domains, we will focus our review by addressing the properties of the APP ectodomain and the intracellular domain. Finally, we provide an update on the current knowledge concerning the APP function in vivo. Understanding the biology of APP is not only interesting from an intellectual point of view, but also of immediate relevance to AD pathogenesis. Because Aβ is generated as part of the normal APP processing, deregulation of Aβ production is expected to simultaneously affect other APP processing metabolites and perhaps APP regulated pathways. As such, perturbation of normal APP physiology may contribute to AD pathogenesis in an Aβ dependent or independent manner. The pathophysiology of Aβ, which has been the focal point of AD research for the past two decades, has been extensively discussed and is beyond the scope of this review.

### I. APP: an overview

#### a) The APP family

**Figure 1:**
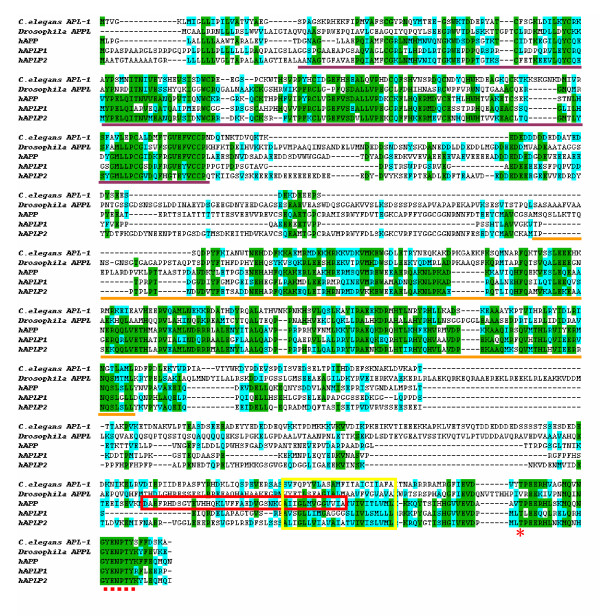
Amino acid sequence alignment of *C. elegans *APL-1, *Drosophila *APPL and human APP, APLP1 and APLP2. Identical sequences are shaded in green and conserved changes in light blue. The E1 and E2 domains are shown in purple and orange underlines respectively. Yellow bracket denotes the transmembrane domain, the Aβ sequences is outlined within the red bracket, and the Thr^668 ^residue and the YENPTY sequence are marked by the asterisk and filled squares underneath the sequences respectively.

APP is a member of a family of conserved type I membrane proteins including APL-1 in *C. elegans *[7], APPL in *Drosophila *[8,9] and APP [10,11], APP like protein 1 (APLP1) [12] and 2 (APLP2) [13,14] in mammals (reviewed in [15]) (Figure 1). These proteins share several conserved motifs, including the E1 and E2 domains in the extracellular sequences and the intracellular domain, the latter exhibits the highest sequence identity. Of interest, the Aβ peptide domain is not conserved and is unique to APP. However, the mammalian APP homologs play redundant activities in vivo (discussed in "The in vivo Function of APP"). The functional conservation of the APP across species is documented by the partial rescue of the *Drosophila APPL *null behavioral phenotype by human *APP *[16]. These observations indicate that the conserved motifs, rather than the Aβ sequence, likely underlie the conserved physiological functions among the APP species.

#### b) APP expression

Mammalian APP family of proteins is abundantly expressed in the brain. Similar to *Drosophila APPL *[17], *APLP1 *expression is restricted to neurons [18]. However, although highly enriched in the brain, *APP *and *APLP2 *can be detected in most other tissues as well. The human *APP *gene, located on the long arm of chromosome 21, spans approximately 240 kb and contains at least 18 exons [19,20]. Alternative splicing generates APP mRNAs encoding several isoforms that range from 365 to 770 amino acid residues. The major Aβ peptide encoding proteins are 695, 751, and 770 amino acids (referred to as APP695, APP751 and APP770). APP751 and APP770 contain a domain homologous to the Kunitz-type serine protease inhibitors (KPI) in the extracellular sequences, and these isoforms are expressed in most tissues examined. APP695 isoform lacks the KPI domain and is predominately expressed in neurons [21]. The reason and functional significance for this apparent tissue-specific alternative splicing is poorly understood.

#### c) APP processing

**Figure 2:**
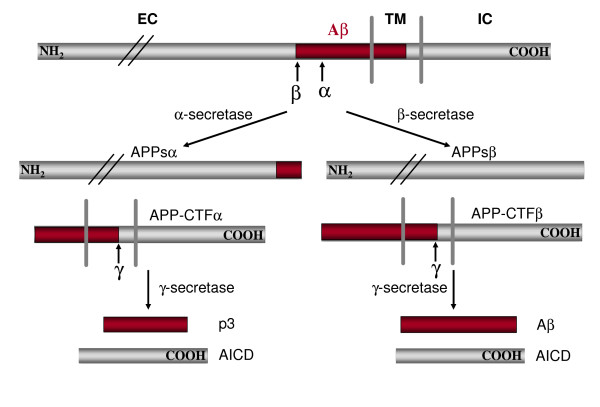
Schematic diagram of APP sequential processing (not drawn in scale). EC: extracellular; TM: transmembrane; IC: intracellular. Aβ domain is highlighted in red. For simplicity, only one cleavage site is shown for each enzyme.

APP undergoes constitutive secretory pathway and is post-translationally modified in route by N- and O-glycosylation, phosphorylation and tyrosine sulphation (reviewed in [2]). Full-length APP is sequentially processed by at least three proteinases termed α-, β- and γ-secretases (Figure 2). Cleavage by α-secretase or β-secretase within the luminal/extracellular domain results in the shedding of nearly the entire ectodomain to yield large soluble APP derivatives (called APPsα and APPsβ respectively) and generation of membrane-tethered α- or β-carboxyl-terminal fragments (CTFs). The major neuronal β-secretase is a transmembrane aspartyl protease, termed BACE1 (β-site APP cleaving enzyme) [22-24]. Several zinc metallopreoteinases, including TACE/ADAM17, ADAM9, ADAM10 and MDC-9, and an aspartyl protease BACE2 can cleave APP at the α-secretase site located within the Aβ domain (Figure 2) (reviewed in [25]), essentially precluding the generation of intact Aβ. Following the extracellular cleavages, γ-secretase processes APP at the carboxyl-terminus of Aβ, producing either a 3 kDa product (p3 in combination with the α-secretase) or Aβ (in concert with BACE1 cleavage), respectively, and the APP intracellular domain (AICD). The γ-secretase activity is primarily executed by a high molecular weight complex containing at least presenilin, nicastrin, anterior pharynx defective (APH1) and presenilin enhancer (PEN2) (reviewed in [6,26]). However, the list of γ-secretase components may be expanding as other new molecules are being identified [27]. These processing events occur in various organelles and also on cell surface. In neurons, APP is anterogradely transported along the axons and is proteolytically processed during transit ([28-30], also discussed below). In non-neuronal cells, APP that reaches to plasma membrane is internalized within minutes through the conserved YENPTY motif (Figure 1) [31]. Following endocytosis, APP is delivered to late endosomes or can be recycled back to cell surface [32].

### II. The APP ectodomain

Various subdomains can be assigned to the APP extracellular sequences based on its primary sequences and structural studies (reviewed in [33,34]). These include the E1 domain, which consists of the N-terminal growth factor-like domain (GFLD) and the metal (copper and zinc) binding motif, the Kunitz-type protease inhibitor (KPI) domain present in APP751 and APP770 isoforms, the E2 domain which include the RERMS sequence and the extracellular matrix components (heparin, collagen, and laminin). We address below the functional studies associated with the APP extracellular domain.

#### a) Cell surface receptor

The analogy of the secondary structures and proteolytic processing profiles between Notch and APP predicts that APP could function as a cell surface receptor similar to Notch (reviewed in [35]). Supporting this hypothesis, Bruce Yankner's group reported that the β-amyloid peptide could bind to APP and thus could be a candidate ligand for APP [36]. Another piece of evidence came from Ho and Sudhof (2004) which showed that APP extracellular domain bind to F-spondin, a neuronally secreted glycoprotein, and this interaction regulates Aβ production and downstream signaling [37]. Similarly, the Nogo-66 receptor was recently shown to interact with the APP ectodomain and by which means affect Aβ production [38]. Finally, the fact that the extracellular domains of the APP family of protein could potentially interact *in trans *(discussed below) suggest that APP molecules can interact in a homophilic manner. However, while the concept is appealing, the evidence that APP could function as a *bona fide *surface receptor remains speculative.

#### b) Cell adhesion

Data linking cell surface APP in cell-substratum and cell-cell adhesion are perhaps more convincing. The E1 and E2 regions have been shown to interact with extracellular matrix proteins and heparan sulfate proteoglycans (reviewed in [39]), supporting its role in cell-substratum adhesion. The same sequences have also been implicated in cell-cell interactions. Specifically, X-ray analysis revealed that the E2 domain of APP could form antiparallel dimers [40]. Such a structure has the potential to function in trans-cellular adhesion. Cell culture studies support the homo- or hetero-dimer formation of the APP family members, and the trans-dimerizations have been shown to promote cell-cell adhesion [41]. However, this activity appears to be mediated by the E1 domain. Downstream of the E1 and E2 regions, a "RHDS" motif in the extracellular domain of APP within the Aβ sequence also appears to promote cell adhesion. It is believed that this region acts in an integrin-like manner by homology to the "RGD" sequence [42]. In this regard, it is interesting that APP colocalizes with integrins on the surface of axons and at sites of adhesion [43,44].

#### c) Neurite outgrowth and synaptogenesis

A neurotrophic and synaptogenic role for APP is perhaps the most consistently documented and arguably the best established, and this function may be linked to its adhesive properties described above. A number of publications have pointed to an important role of the APP extracellular domain in this activity, both as a full-length protein and as a secreted molecule (APPs) following ectodomain shredding. Thus, APP may exert these activities in both autocrine and paracrine fashions. APP expression is upregulated during neuronal maturation and differentiation [45,46]. Its expression is also induced during traumatic brain injury both in mammals and in *Drosophila *[47-50]. APP undergoes rapid anterograde transport and is targeted to the synaptic sites [21,29,51,52], where the levels of secreted APP coincide with synaptogenesis [53].

The crystal structure of the E1 domain shows similarities to known cysteine-rich growth factors that it is thus termed as the growth factor-like domain (GFLD) [54]. One of the earliest indications of APP function came from the observation that assessing fibroblasts treated with an antisense *APP *construct grew slower and the growth retardation can be restored by treatment with secreted APPs [55]. The active domain was subsequently mapped to a pentapeptide motif "RERMS" in the E2 domain [56]. The activity is not limited to fibroblasts as infusion of this pentapeptide or APPs into brain resulted in increased synaptic density and improved memory retention in animals [57,58], while injection of APP antibodies directly into the brain led to impairment in behavioral tasks in adult rat [57]. This finding is corroborated by reports showing that reduction of APP is associated with impaired neurite outgrowth and neuronal viability in vitro and synaptic activity in vivo [59-61]. A recent paper further substantiated this finding, showing that the growth promoting property is mediated by the ability of APPsα to down-regulate CDK5 and inhibit tau hyperphosphorylation [62]. Finally, Caille et al. indicated the presence of binding sites for APPs in epidermal growth factor (EGF)-responsive neural stem cells in the subventricular zone in the adult rodent brain [63]. In this context, APPsα acts as a co-factor with EGF to stimulate the proliferation of these cells both in neurospheres in culture and in vivo. Because APPs levels have been reported to be reduced in individuals with AD [64], this result raises the possibility that the loss of the trophic activity of APPs, in concert with the reduction of other growth factors in the brain, may contribute to the neurodegeneration in AD.

### III. The APP intracellular domain

The high degree of sequence conservation between the intracellular domain of APP, APLP1, and APLP2 predicts that it is a critical domain regulating APP function. Indeed, multiple important roles have been assigned to this domain, most notably in axonal transport and cell signaling.

#### a) Phosphorylation and protein-protein interaction

APP can be phosphorylated at multiple sites in both extracellular and intracellular domains [65]. Among these, the phosphorylation at the threonine residue (Thr^668^) in APP intracellular domain (Figure 1) has received most of the attention to date. Several kinases have been implicated in this phosphorylation event, including the cyclin-dependent kinase 5 (CDK5), c-Jun N-terminal kinase 3 (JNK3), and GSK3β [66-68]. This phosphorylation has been implicated to regulate APP localization to the growth cones and neurites [68,69]. Significantly, the Thr^668 ^phosphorylated APP is shown to be preferentially transported to the nerve terminals [70], and that the Thr^668 ^phosphorylated APP fragments are increased in AD, but not in control subjects [71], raising the possibility that this phosphorylation event may contribute to AD pathogenesis by regulating Aβ generation in neurons. A recent work provided a possible link between Thr^668 ^phosphorylation and APP processing through the prolyl isomerase Pin1. The authors showed that Pin1 binds to the phosphorylated Thr^668^-Pro motif and promotes the isomerization of the proline residue [72]. This leads to the conformational change of the APP intracellular domain and alteration of APP processing and Aβ production.

In addition to Thr^668 ^phosphorylation, the highly conserved APP intracellular domain has been shown to bind to numerous proteins (reviewed in [73,74]). Of particular interest and relevance to this review, the YENPTY motif is required to interact with various adaptor proteins, including Mint-1/X11a (and the family members Mint-2 and Mint-3, so named for their ability to interact with Munc18), Fe65 (as well as Fe65 like proteins Fe65L1 and Fe65L2) and c-Jun N-terminal kinase (JNK)-interacting protein (JIP), through the phosphotyrosine-binding (PTB) domain. Interestingly, the APP Thr^668 ^phosphorylation and adaptor protein interaction may be functionally coupled. Biochemical and structural studies indicate that Thr^668 ^phosphorylation results in a conformational change which negatively regulates APP binding to Fe65 and reduces the stability of the APP intracellular domain [67,75,76]. This phosphorylation has also been reported to modulate APP interaction with Mint-1/X11a [77]. Overall, these findings lend support for an important role of Thr^668 ^phosphorylation and Fe65 and/or Mint-1/X11a in regulating APP dynamics. Of interest is the finding that Fe65 acts as a functional linker between APP and LRP (another type I membrane protein containing two NPXY endocytosis motifs) in modulating endocytic APP trafficking and amyloidogenic processing [78].

#### b) Cell migration and synapse remodeling

In addition to stabilizing AICD and modulating nuclear signaling, the binding of APP with Fe65 has been implicated in regulating cell motility and growth cone dynamics [79,80]. In H4 neuroglioma and MDCK cells, APP is found to associate with Fe65 and mammalian homolog of Enabled (Mena), a cytoskeletal protein expressed in active actin remodeling areas such as lamellipodia and growth cones (reviewed in [81]). The functional role of this complex is documented in a wound healing assay in MDCK cells in which the authors showed increased rate of cell migration and wound closure by overexpressing APP and Fe65, and this process appears to be partially dependent on Mena/actin complex [79]. Subsequent analysis by the same group showed that in primary neurons, APP and Fe65 are concentrated in actin-rich area of the growth cones, supporting an active role of the APP/Fe65 complex in growth cone dynamics and synapse remodeling [80]. Indeed, a physiological significance of the APP/Fe65 interaction is supported by loss-of-function studies in mice and in *C. elegans *(discussed in "The in vivo Function of APP).

APP also interacts with Mint/X11 family of proteins through the same YENPTY motif. Using *Drosophila *as a model system, it was shown that APPL was required to facilitate cell adhesion molecule fasciclin-mediated synapse formation at *Drosophila *neuromuscular junction, and this process involved the interaction with dMint [82]. How these adaptor proteins are coordinated to contribute to various APP processes is not clear and needs to be further studied.

#### c) Cell signaling

In addition to the γ-cleavage that yields Aβ 40 and Aβ 42, PS-dependent proteolysis also occurs at other positions including the ε-site (Aβ 49) downstream of the γ-site proximal to the membrane-intracellular boundary [83-85]. Recent data provide support for a sequential cleavage model in which ε-cleavage serves as the initial cutting site followed by δ- and γ-processing within the membrane [85-87]. Thus the ε-cleavage of APP may represent the primary PS-dependent processing event. This is important because this cleavage releases an APP intracellular domain (AICD) that is highly reminiscent of the release of the Notch intracellular domain (NICD) after γ-secretase processing, the latter being an obligatory step in Notch mediated signaling (reviewed in [35]). Accordingly, PS-dependent AICD has been shown to translocate to nucleus and could function as a transcriptional regulator [88-90]. AICD is very labile but can be stabilized by Fe65 [88]. Using a heterologous reporter system, AICD is shown to form a transcriptionally active complex presumably in the nucleus together with Fe65 and Tip60 [89,91]. However, subsequent analyses have suggested that the earlier view may be too simplistic and incomplete. First, follow up studies by Cao et al. showed that nuclear translocation of AICD is not required but may be indirect through Fe65 [92]; secondly, PS-dependent AICD production is not required for the APP signaling activity as it proceeds normally in PS null cells and by PS inhibitor treatment [93]. Instead, the authors provide an alternative pathway for this activity that involves Tip60 phosphorylation. Lastly, a recent report documented that the proposed signaling activity is executed by Fe65 and that APP is not required altogether [94]. Overall, a potential nuclear signaling activity remains to be established.

In spite of the unclear nature of how AICD may activate signaling pathways, a trans-activating role of the APP/Fe65/Tip60 complex has been consistently documented, at least in overexpression systems. Accordingly effort has been taken to identify the downstream targets. Two genes have been proposed to date, KAI1, a tumor suppressor gene, and neprilysin, a neutral endopeptidase with Aβ degrading activity [95,96]. The latter pathway is particularly interesting because it suggests that γ-secretase release of AICD can regulate the degradation of Aβ in the extracellular space. If this is true, it will be important to know the feedback pathways that modulate γ-secretase activity to regulate neprilysin expression. The proposed AICD signaling has also been implicated in phosphoinositide-mediated calcium signaling and cell cycleregulation ([97] and reviewed in [98]). Specifically, fibroblast cells lacking APP exhibit calcium signaling defect which can be rescued by expressing AICD, and the aforementioned CDK5 and Pin1 that interact and regulate the APP intracellular domain are linked to cell cycle events.

#### d) Apoptosis

The preceding sections have highlighted the positive or beneficial functions of APP. Interestingly, there is a rather lengthy history of cytotoxic properties of APP, especially when APP or the β-cleaved C-terminal fragment of APP ("C99" or "C100") are overexpressed [99,100]. Indeed, overexpression of the C100 APP C-terminal fragment (CTF) is associated with neuronal degeneration in brain [101], perhaps by perturbing APP signal transduction. Another pathway by which the APP CTF is cytotoxic may be through AICD. Specifically, the cytotoxicity of APP CTF appears to require an intact caspase site within the cytosolic tail [102]. In this cell culture model, loss of this caspase site by mutating the aspartate residue at position 664 to alanine (D664A) resulted in a loss of C100 associated cytotoxicity. It has been proposed that release of the smaller fragments (C31 and Jcasp) from AICD after cleavage at position 664 results in the generation of new cytotoxic APP related peptides [103]. Indeed, in an APP transgenic mouse line in which the caspase site is mutated to render APP noncleavable, the predicted Aβ-related phenotypes in brain, including synaptic, behavior, and electrophysiological abnormalities, were absent in spite of abundant amyloid deposits in brain [104]. Therefore, release of the smaller fragments (C31 or Jcasp) after caspase cleavage of C99 may result in activation of genes that contribute to cell death in a manner independent of γ-secretase. Therefore, there are at present several potential mechanisms whereby APP may contribute to neurotoxicity: via γ-secretase cleavage to release AICD or via alternative cleavage of the APP C-terminus to release other cytotoxic peptides.

#### e) Axonal transport

The neuron is unique in cellular morphology with a long axon and a rich dendritic arbor. Elaborate protein trafficking exists in neurons for selected proteins to reach their designated compartments and to be transported back to the cell bodies. Protein processing and modifications are known to take place during the transit in axons. APP is transported in axons via the fast anterograde transport machinery, a process that requires kinesin molecular motors, and that at least one documented source of amyloid deposits originates from synaptically released Aβ pool [105,106]. The anterograde transport of APP is proposed to be mediated by binding of APP with kinesin light chain (KLC) subunit, a component of the kinesin-1 transport machinery [107]. However, recent evidence is more consistent with the view that the interaction is mediated indirectly through adaptor proteins, of which JIP-1, a member of the JNK-interacting protein family (JIP), is a likely candidate as it is known to interact with both KLC and APP [108]. The fact that either deletion or overexpression of the *Drosophila *APP homolog, APPL, in *Drosophila *neurons disrupts axonal transport, a phenotype similar to that seen in flies lacking components of the kinesin motor [109,110], prompted Goldstein and colleagues to propose that APP may represent a kinesin cargo receptor, linking kinesin-1 to a unique subset of transport cargos. This model is consistent with the observation that the cargos that carry APP anterogradely in axons are different from the transport carrier of synaptophysin [111].

Unresolved by this model is how APP is initially sorted into a particular class of vesicles. The potential importance of the initial sorting of APP is underscored by the report that BACE1 and presenilins are contained within the same kinesin-1 dependent APP transport vesicles [30]. This finding led to the suggestion that not only is APP required for the delivery of the enzymatic machinery necessary for Aβ production, but Aβ generation also occurs enroute from the cell body to the nerve terminals within the transport cargo that is carried by APP. However, the report that APP is a kinesin-1 receptor and a common vesicular compartment carried all the processing machinery necessary for Aβ generation have not been confirmed by others [112]. Nevertheless, KLC deficient animals, when crossed with APP transgenic mice, showed axonal pathology manifested by axonal swellings and increased amyloid levels and deposits in brain [113]. The latter argue that perturbations of axonal transport during aging may predispose to the development of AD pathology.

### IV. In vivo function of APP

The in vivo gain- and loss-of-function phenotypes associated with the APP family of proteins in model systems (*C. elegans*, *Drosophila *and mice) are consistent with a role of APP in neuronal and synaptic function in both central and peripheral nervous systems. This may involve the APP ectodomain, the APP intracellular domain, the Aβ sequence or, indeed, the cross communications among these motifs. These findings will be discussed next in the respective animal models.

#### a) C. elegans

Although the cloning of the *C. elegans APP *homolog *APL-1 *was published in 1993 [7], to date there has been no report describing the *APL-1 *null mutant. Using RNA-interference (RNAi) method, Zambrano et al. reported that worms treated with *APL-1 *RNAi exhibit defect in pharyngeal pumping and, interestingly, this phenotype is shared with Fe65 homolog-1 (Feh-1) treated worms [114], suggesting that these two proteins act in the same pathway possibly through direct physical interactions. Of note, the *C. elegans *pharynx is a cholinergic neuromuscular structure that uses acetylcholine as a neurotransmitter. A possible role of the APL-1/Feh-1 complex in regulating the cholinergic function is provided by the same group showing that mutation in Feh-1 results in a reduction of acetylcholinesterase gene expression [115]. Because our own studies document an indispensable activity of the APP family of proteins in regulating cholinergic neurotransmission at the neuromuscular junction (NMJ) ([116], and see below), these findings taken together support a conserved role of APP family of proteins in cholinergic pathway possibly in a Fe65 dependent manner.

#### b) Drosophila

*Drosophila *mutants lacking APPL are viable, fertile but exhibit subtle behavioral defects that can be partially rescued by human *APP*, demonstrating functional conservation [16]. Subsequent analysis revealed that these mutant flies show reduced synaptic bouton numbers at neuromuscular junction [117], and that this activity appears to require the formation of a complex with the cell adhesion molecule fasciclin and *Drosophila *Mint/X11 [82]. Consistent with a role of APPL in synapse development, ectopic overexpression of *APPL *leads to satellite bouton formation at *Drosophila *NMJ, and this activity requires the APP YENPTY domain where adaptor protein interaction takes place [117].

Similar gain-of-function studies in *Drosophila *revealed a spectrum of other phenotypes, ranging from a blistered wing phenotype that may involve cell adhesion [118], a Notch gain-of-function phenotype in mechano-sensory organs, which reveals a possible genetic interaction of APP and Notch through Numb [119], and a neurite outgrowth phenotype that is linked to the Abelson tyrosine kinase and JNK stress kinase [50]. Although the pathways implicated in each of the phenotypes are distinct, they all seem to require the APP intracellular domains and protein-protein interactions through the conserved YENPTY sequence. These ectopic overexpression studies should be interpreted with caution though as APP interacts with numerous adaptor proteins through the YENPTY motif and many of the APP binding partners also interact with other proteins. Therefore, the phenotypes observed by overexpressing *APP *or *APPL *could be caused by the disturbance of a global protein-protein interaction network. Interestingly, similar to the mammalian system, APPL is found to be upregulated in traumatic brain injury [50], supporting a potential activity of APP family of proteins in nerve injury response and repair. However, the functional significance of this nerve injury response remains poorly understood.

#### c) Mice

To understand the in vivo function of APP and its processing products, we generated an *APP *null mutation in mice [120]. Homozygous *APP *deficient mice are viable and fertile. However, the mutant animals were smaller (15–20% less body weight) than age-matched controls and exhibited decreased locomotor activity and forelimb grip strength, indicating compromised neuronal or muscular function. In addition, the majority of the mice show reactive gliosis, suggesting undefined neuronal damage in brain activity. Indeed, subsequent analysis reveal that these mice exhibit behavioral deficit in Morris water maze task [121,122], and are also defective in long-term potentiation and GABA-mediated postsynaptic response [123]. However, these impairments were not caused by a gross loss of neurons or synapses because unbiased stereological quantification failed to detect any loss of neurons or synaptic bouton numbers in aged *APP *null mice [122]. The relatively subtle phenotype of the *APP *deficient mice indicated that the presence of other APP family members may compensate for the loss of APP. Indeed, while the *APLP1 *and *APLP2 *single null mice are also viable and fertile, *APP/APLP2*, *APLP1/APLP2 *double knockout mice showed early postnatal lethality [124,125]. Intriguingly, the *APP/APLP1 *double null mice are viable [125], revealing a property of APLP2 that is uniquely required when APP or APLP1 is absent.

In the peripheral nervous system, *APP*/*APLP2 *double knockout animals exhibited poorly formed neuromuscular junction (NMJ) with reduced apposition of pre- and postsynaptic elements of the junctional synapses [116]. The number of synaptic vesicles at the presynaptic terminals were also reduced, a finding confirmed by defective neurotransmitter release. With knowledge of the NMJ phenotypes of *APP*/*APLP2 *double knockout mice in mind, examination of the parasympathetic submandibular ganglia of these animals also showed a reduction in active zone size, synaptic vesicle density, and number of docked vesicles per active zone [126]. The NMJ phenotypes in *APP/APLP2 *null mice are in some aspect similar but in others distinct from *APPL *null *Drosophila*, as the latter exhibits a subtle reduction of synaptic bouton numbers without structural alterations [117]. Furthermore, while the major synaptic phenotype can be seen when *APPL *is overexpressed [82,117], no NMJ defect can be detected in transgenic mice overexpressing human *APP *(HZ, unpublished data).

Deficiency of all three *APP *genes led to death shortly after birth. The majority of the animals showed cortical dysplasia, suggestive of migrational abnormalities of the neuroblasts and partial loss of cortical Cajal Retzius cells [127]. Interestingly, this defect is phenocopied in mice doubly deficient in APP binding proteins Fe65 and Fe65L1 [128]. This result is consistent with the aforementioned *C. elegans *studies, all supporting a common physiological activity of the APP/Fe65 complex. It should be pointed out, however, that morphological similarity does not necessarily implicate functional interaction. Indeed, cortical dysplasia with variable penetrance also exists in mice deficient in various other proteins including PS1, β1 and α6 integrins, focal adhesion kinase, α-dystroglycan and laminin α2 (reviewed in [129]).

Taken together, the recent findings presented a convincing picture that members of the *APP *gene family play essential roles in the development of the nervous system relating to synapse structure and function, as well as in neuronal migration or adhesion. Whether these abnormalities underlie the early postnatal survival of the animals remain to be established. Further, whether these activities are due to mechanical properties or mediated by activating signaling pathways, or both, are interesting questions that remain to be elucidated.

## Concluding remark

This review has examined some of the physiological functions of APP, which has so far met with a great deal of excitement and scientific challenge. It is clear that APP undergoes complex regulation and is important for neuronal and synaptic function. Since genetic mutations in *APP *are causal for AD, and abnormal expression of wild-type APP are linked to AD pathology in Down Syndrome patients [130] and early-onset AD with cerebral amyloid angiopathy [131]), it is reasonable to speculate that disturbance of APP-regulated pathways may directly contribute to neuronal and synaptic impairment and disease pathogenesis in an Aβ-dependent or Aβ-independent manner. As such, a more complete understanding of AD pathogenesis will likely require greater insights into the physiological functions of APP.

## Abbreviations

AD, Alzheimer's disease; APP: amyloid precursor protein; APLP1: APP-like protein 1; APLP2: APP-like protein 2; Aβ, β-amyloid; EC: extracellular; IC: intracellular; TM: transmembrane; AICD: APP intracellular domain; CTF: carboxyl terminal fragment; KLC: kinesin light chain; NMJ: neuromuscular junction.
